# Development, Feasibility, Acceptability, and Usability of an Artificial Intelligence–Powered Chatbot (Suzy) to Support Patients in Substance Use Disorder Recovery: Multiphase Study

**DOI:** 10.2196/84683

**Published:** 2026-05-20

**Authors:** Warren Scott Comulada, Dallas Swendeman, Y. Xian Ho, Joanna M Streck, Delta-Marie Lewis, Roxana Rezai, David Warren, Gladys Pachas, Maria Chandler, Jonathan L Jackson, Lillian Gelberg

**Affiliations:** 1 Department of Psychiatry and Biobehavioral Sciences David Geffen School of Medicine University of California, Los Angeles Los Angeles, CA United States; 2 Department of Health Policy and Management Fielding School of Public Health University of California, Los Angeles Los Angeles, CA United States; 3 Department of Epidemiology Fielding School of Public Health University of California, Los Angeles Los Angeles, CA United States; 4 Dimagi, Inc Cambridge, MA United States; 5 Center for Addiction Medicine Department of Psychiatry Massachusetts General Hospital/Harvard Medical School Boston, MA United States; 6 Tobacco Research and Treatment Center Division of General Internal Medicine Massachusetts General Hospital/Harvard Medical School Boston, MA United States; 7 TCC Family Health aka The Children's Clinic Long Beach, CA United States; 8 Department of Family Medicine David Geffen School of Medicine University of California, Los Angeles Los Angeles, CA United States

**Keywords:** substance use disorder, peer recovery coach, health coach, artificial intelligence, chatbot, large language model, GPT, iterative development, human-centered design

## Abstract

**Background:**

Substance use disorder (SUD) remains a major public health crisis in the United States, with significant challenges in treatment access, retention, and workforce capacity. SUD care teams, including addiction medicine physicians and peer recovery coaches (PRCs), support patients receiving SUD treatment but face heavy workloads and burnout. Artificial intelligence (AI) innovations, particularly large language model (LLM)–based chatbots, may extend PRC support and provide patients with on-demand recovery support between clinic visits and PRC contacts. However, evidence on their development, feasibility, acceptability, and usability in addiction services remains limited.

**Objective:**

This study describes the development, feasibility, acceptability, and usability of an AI-powered health coaching chatbot (Suzy) designed to support patients in SUD recovery.

**Methods:**

A total of 2 clinicians, 5 researchers, and 2 technology developers led a small, multiphase pilot study. In the formative phase, they conducted focus groups and qualitative in-depth interviews with 12 health care professionals and 8 patients with substance use histories to specify chatbot functions and develop a rule-based chatbot. In phase 2, they conducted usability testing of the rule-based chatbot with 8 patients who reported substance use and completed standardized tasks, surveys, and qualitative interviews. Measures included the System Usability Scale (SUS), Net Promoter Score (NPS), and Single Ease of Use Question (SEQ). In phase 3, they developed an LLM-based chatbot co-designed and fine-tuned with PRCs and other SUD experts.

**Results:**

Rule-based chatbot functions included craving management, appointment reminders, resource referrals, care team contacts, and goal setting. Usability task testing supported feasibility. In this small pilot sample, quantitative and qualitative feedback indicated acceptability and usability, with an average SUS score of 93 (benchmark 68), an NPS of 63 (benchmark 35), and a mean SEQ score of 6.5/7. Patients valued Suzy’s approachable, nonjudgmental language and features that promoted accountability, self-monitoring, and 24/7 availability, while emphasizing that chatbots should supplement but not replace human support. The LLM-based chatbot development emphasized information accuracy, safety escalation protocols to mitigate risks of inappropriate chatbot responses, human-in-the-loop features, and expanded conversational flexibility and personal tailoring.

**Conclusions:**

In this pilot study, a rule-based chatbot designed to support SUD care demonstrated feasibility, usability, and acceptability. LLM-based chatbot development required more robust safety and emergency reporting features, while offering more patient-responsive conversational functions. By providing on-demand coaching, referrals, and reminders, Suzy may extend the reach of care teams, alleviate provider burden, and enhance patient engagement. Additional work is needed to understand how to best integrate Suzy into patients’ recovery journeys to ensure human support remains accessible and prioritized. LLM evaluation was based on expert testing and safety review. Clinical effectiveness, including the impact on substance use, was not evaluated. Next steps include evaluating the LLM chatbot in real-world settings with larger samples and assessing its efficacy in reducing substance use.

## Introduction

Substance use disorder (SUD) remains a costly public health crisis in the United States, accounting for an estimated 80,391 annual overdose deaths in 2024, including 54,743 overdose deaths involving opioids [1]. SUD generated over US $13 billion in emergency department and inpatient care costs in 2017 [2]. Sustained engagement with a clinical care team is critical to an individual’s SUD recovery. Health care facilities and SUD treatment centers have moved toward a chronic care approach, with an emphasis on longitudinal and multidisciplinary treatment services [3], which often include addiction medicine or primary care physicians who provide medication treatment for SUD, behavioral health providers (eg, psychiatrists and social workers), and peer recovery coaches (PRCs) who leverage lived experience with addiction to provide nonclinical support coaching [4]. These teams aim to support longer-term engagement and remission outcomes in SUD care [5,6].

However, SUD treatment programs face challenges in achieving economies of scale, including shortages of and burnout among addiction medicine specialists, PRCs, social workers, and other clinicians, along with limited time to adequately address SUD treatment [7,8]. These factors contribute to low uptake and retention in SUD treatment. In 2021, only 6% of people aged 12 years or older with SUD received treatment [9]. In a study of patients with SUD at a low-threshold bridge clinic from 2016 to 2021, 70% initiated care, but only 38% were retained at 2 months [10].

Challenges in SUD treatment programs are driving paradigm shifts in addiction medicine staffing, leading to task shifting to paraprofessional and peer workers. PRCs are individuals with lived SUD recovery experience who are trained to provide ongoing support to patients in SUD treatment [11,12]. In recognition of the value of persons with lived experience and the Collaborative Care Model [13], treatment programs are integrating PRCs into their multidisciplinary teams [4,11,12]. Converging data indicate that PRCs improve treatment retention, promote long-term substance use abstinence, and reduce stigma around medications for SUD [4,11,14,15]. The contribution of PRCs is increasingly recognized by public payers, such as Medicaid, which has begun covering peer support services and accelerating their adoption in SUD treatment programs [16]. While helping reduce clinicians’ workload, PRCs also report feeling overworked and emotionally drained, with up to two-thirds reporting levels of exhaustion indicative of burnout [17]. This underscores a critical need for low-cost solutions to support these role groups and their patients in SUD recovery.

Novel digital health technologies, such as artificial intelligence (AI) innovations, are emerging as potential solutions to support PRCs, other staff, and their patients, but more research is needed to validate promising AI developments for SUD care [18,19]. A previous study showed that implementation of an AI-based screening tool for SUD risk was associated with a reduction in 30-day readmissions and cost savings of up to US $6801 per avoided readmission [20]. Researchers also piloted an AI-based chatbot to screen for SUD, anxiety, and depression and to refer patients to a statewide SUD prevention, screening, and treatment program; the chatbot was appealing and acceptable to study participants [21]. The emergence of large language models (LLMs) has expanded chatbot capabilities to provide health coaching interventions by mimicking human conversation better than earlier rule-based chatbots and AI deployments [22-24]. Studies have demonstrated the acceptability of chatbot-based interventions [25,26] and their efficacy in reducing substance use in a randomized controlled trial [24]. Potential benefits reported by peer health coaches, their supervisors, and clients participating in HIV prevention and treatment intervention studies indicate that chatbots can support coaches’ work by assisting with referrals to services and resources, appointment scheduling and reminders, routine check-ins, and overall reliability and consistency of availability and responsiveness, thus reducing work-related stress and hassles and improving quality of care [27].

While chatbots are promising intervention tools, further studies are needed to understand their optimal use, risks, and benefits in addiction medicine settings. Drawing lessons from their application in mental health care, seemingly positive chatbot features, such as humanlike interactions and 24/7 availability, may impart unintended consequences [28]; patients may develop unhealthy attachments to chatbots and make inquiries related to suicidal ideation and other topics that require human intervention after hours, when a human counselor is not available. In addition to suicide risk, addiction medicine safety protocols are complicated by the need to address SUD detoxification, overdose risk, and withdrawal symptoms requiring medication-related decision-making that carries safety implications, such as buprenorphine dosing [29]. These situations require more conservative protocols for safety routing to direct patients to seek medical attention rather than dispense medical advice. Unlike mental health conditions, SUD may involve legal consequences, raising concerns about how chatbots respond to queries about substance acquisition or concealment. Chatbots in addiction medicine settings need to operate within multidisciplinary, team-based workflows that include PRCs, psychiatrists, psychologists, and other clinicians, to complement rather than impede their work. This contrasts with stand-alone chats that may suffice to address lower-acuity mental health conditions. Stigma toward SUD, a known barrier to treatment engagement, can be embedded in chatbot knowledge bases and manifest during chatbot conversations with patients [30]. Stigma intersects with cultural and social factors that also impact chatbot engagement, such as the ability of a chatbot to establish rapport with patients [31].

LLM-based chatbots are subject to novel challenges relative to rule-based chatbots, including “hallucinations,” where LLMs fabricate information or make incorrect or inappropriate recommendations; biases that can seed chatbot responses with inappropriate and discriminatory language; and safety and privacy concerns [32,33]. LLM-based chatbots also offer opportunities to better mitigate adoption and implementation barriers relative to their rule-based counterparts, for example, by minimizing stigmatizing language relative to human-written content [34]. AI algorithms that predict risk of adverse treatment outcomes [35], in concert with LLM-based chatbots tailored for target populations [36], could improve patient adoption and treatment.

This pilot study with a small sample explores the feasibility, acceptability, and usability of a rule-based chatbot and a subsequent LLM-based chatbot designed to support patients with SUD as a health coach (Suzy), serving between contacts with their PRCs and SUD treatment visits. Suzy’s role addresses several converging needs in addiction medicine, including the provision of timely encouragement, reminders, and service referrals for patients when they experience cravings and stressors outside of clinic hours. Suzy’s role also has the potential to extend the reach of PRCs and other support staff without requiring additional staffing, thereby alleviating addiction medicine staffing shortages and burnout. We describe the Suzy development process in a clinical setting, risks and concerns given the capabilities and limitations of AI-driven chatbots, and the guardrails employed. The goal of this early-stage work is to inform health care teams providing addiction services of important considerations when determining how best to integrate chatbots into their own clinical practice. This is done in the context of our own chatbot development process, which pivoted from a scripted (rule-based) chatbot to an LLM-based chatbot in light of recent LLM advances and commercial availability. Based on the acceptability of chatbots in other clinical studies, we hypothesize that the chatbots will be feasible, acceptable, and usable to meet the health coaching needs of patients with SUD.

## Methods

### Overview

The study team was led by 2 clinicians, 5 researchers, and 2 technology developers from 3 institutions, with expertise in addiction medicine, primary care, computer and implementation science, health coaching, digital health intervention development, and AI. Team members at Dimagi Inc, a small-business social enterprise that specializes in health care technology, developed a rule-based and then an LLM-driven chatbot for patients in office-based treatment for SUD to access between care team contacts. Team members at Harvard Medical School, Massachusetts General Hospital, and the University of California, Los Angeles collaborated with Dimagi Inc to provide subject matter expertise to inform chatbot development, carry out participant recruitment, conduct chatbot evaluation, and perform other research activities. The study team designed chatbot functions to support long-term follow-up care immediately following substance use screening for moderate- to high-risk SUD. The chatbot provided referrals and aided patients in using strategies to handle substance use cravings and stress, similar to guidance that would be provided by a PRC. This pilot study consisted of 3 phases: (1) a formative phase that obtained user input, which then guided the design and development of a rule-based chatbot and its core functions; (2) a design validation phase in which the feasibility and acceptability of the rule-based chatbot were assessed via usability testing with potential target end users; and (3) a development phase to convert the rule-based chatbot into an LLM-driven chatbot. This paper follows reporting guidelines from the TRIPOD (Transparent Reporting of a multivariable prediction model for Individual Prognosis Or Diagnosis)-LLM (Multimedia Appendix 1) [37].

### Ethics Approval

Institutional review board (IRB) approval was obtained from the New England Review Board, Massachusetts, USA (approval number 120190532, now Western Institutional Review Board-Copernicus Group) for the first 2 study phases. The IRB determined that the study posed minimal risk and approved verbal consent for participation. A study team member trained in human research obtained verbal consent from participants. Phase 3 did not include study participants; phase 3 data consist of feedback from PRCs and study team members who served in an advisory role.

### Phase 1: Formative Work

#### Participants

In the formative phase, the study team conducted individual and group interviews with health care professionals and adult primary care patients with substance use experience. A total of 12 health care professionals participated in interviews from January to July 2021. The team conducted 4 focus groups comprising 5 health coaches, 3 primary care physicians, 1 behavioral health specialist, 1 social worker, 1 health education executive, and 1 medical officer. The focus group format was used to facilitate discussion among participants from different professions. Separate qualitative, in-depth, semistructured 1-on-1 interviews were conducted with 2 health professionals due to scheduling difficulties for focus groups. The team purposively sampled health care professionals affiliated with TCC Family Health, a federally qualified health center in Los Angeles County, California, through their professional networks. Eligible professionals were at least 18 years old.

The study team also conducted separate in-depth interviews with 8 adult primary care patients recruited through recruitment flyers, provider referrals, and email invitations from the federally qualified health center, as well as through snowball recruitment and an observational cohort study assessing the epidemiological impact of substance use and HIV [38]. Eligible patients were at least 18 years old, receiving primary care in California, had SUD, or reported recent use of substances other than alcohol, tobacco, and cannabis. The study team deemed sample sizes of 8 patients and 12 health care professionals to be sufficient based on prior research [39,40]. Data saturation is often reached within the first 12 participants, especially in studies with focused objectives and smaller sample sizes, such as this one. Qualitative findings informed chatbot functionality.

#### Data Collection

Two of the authors (YXH and DML) conducted focus groups and in-depth, 1-on-1 interviews following a semistructured format using a virtual conferencing platform. In this semistructured approach, moderators (authors) followed a predefined interview guide with open-ended questions and probes while remaining flexible in allowing participants to discuss new topics. This approach ensured consistency across interviews while allowing participants to elaborate on their experiences with SUD care and provide nuanced feedback to aid chatbot development. The moderators have extensive prior experience facilitating qualitative data collection and analyses of digital health interventions. The study team developed the interview guide specifically for this study based on their clinical experience with patients with SUD and research background in developing other AI tools, and iteratively refined the guide questions to align with the study objectives. The guide was designed to elicit participants’ perspectives on experiences with drug screening and care, barriers to SUD care and substance use reduction, the potential use of chatbot technology to support patient needs between office-based treatment visits, and end-user acceptability of a chatbot. The study team audio-recorded focus group and interview sessions with participant consent.

#### Data Analysis

Audio recordings of focus groups and interviews collected during the formative phase and usability testing sessions were transcribed by a HIPAA (Health Insurance Portability and Accountability Act)–compliant transcription service. Deidentified transcripts were analyzed using qualitative content analysis [41]. One of the authors (DML) first used deductive coding corresponding to a priori themes based on interview probes to identify common barriers to SUD recovery, recommendations for chatbot design, and potential facilitators and barriers to chatbot implementation. A second author (YXH) then reviewed the codes, which were discussed and refined iteratively to mitigate bias and ensure agreement. Other research team members subsequently reviewed the codes to incorporate multidisciplinary perspectives, interpret findings, and ensure alignment between themes and the raw qualitative data. To address reflexivity [42], YXH and DML acknowledged their professional backgrounds in digital health and behavioral research. They engaged in regular discussions with team members throughout the qualitative analysis process to examine how prior assumptions about chatbot utility and SUD care were influencing their interpretation of the qualitative data. This approach helped mitigate potential biases in interpreting qualitative data.

#### Rule-Based Chatbot Development

**Figure 1:**
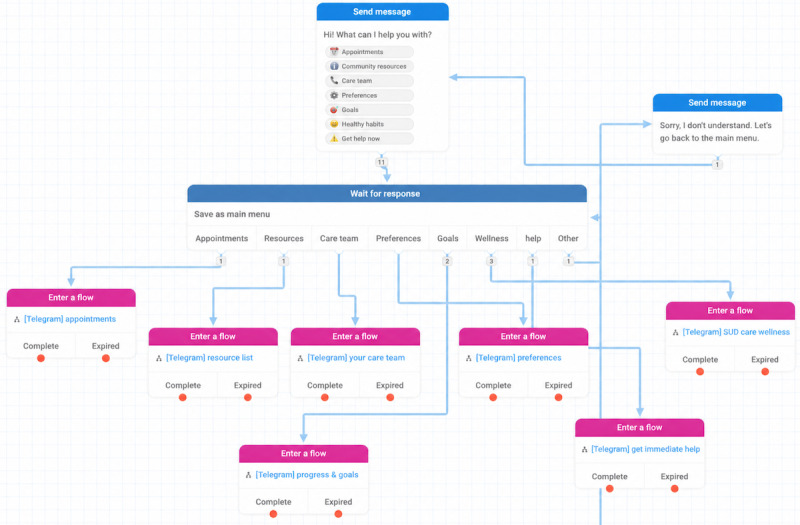
Architecture of the original rule-based chatbot. Users either select response options from a menu or enter free text. A natural language processing algorithm detects keywords or key phrases in the input and routes the dialogue accordingly between the user and the chatbot. SUD: substance use disorder.

Study team members iteratively designed Suzy, a rule-based chatbot [43]. Suzy used flow-based logic trees developed by the study team using TextIt, a chatbot-building platform for designing interactive messaging workflows (Figure 1). Suzy delivered messages via the Telegram instant messaging platform (Telegram FZ-LLC). Users interacted with Suzy by selecting from a menu of button responses or entering text queries and responses on their smartphones, as they would for an SMS text message. The study team selected Telegram to support rapid prototyping and real-world usability testing of a chatbot that did not require participants to download or learn to use a new, study-specific app.

### Phase 2: Feasibility, Acceptability, and Usability Testing

#### Participants

The team recruited 8 participants (6 new patients and 2 patients from the phase 1 formative work) for usability testing sessions. The study team used the same recruitment methods and eligibility criteria as those used for patients in phase 1. A sample size of 8 patients was determined to sufficiently capture most usability issues, based on prior studies demonstrating that sample sizes as small as 5 participants may suffice [39,44,45].

#### Data Collection

The study team created 12 usability tasks that patients would commonly perform using Suzy outside of office-based treatment visits, based on formative findings. An example of one of the tasks is shown below:

- Scenario: You would like to see what support groups are in your area.
- Task: Check in with Suzy about support groups available to you. Identify the name of a support group on the list.

An experienced moderator and research associate (YXH and DML) coled individual, 1-hour, remote, moderated usability testing sessions with participants in June 2022. Consent was obtained at the start of each 1-hour session, after which participants tested the chatbot, completed an acceptability and usability survey, and participated in an interview to provide feedback on their experience using Suzy. Participants responded to open-ended questions regarding their perceptions of their interaction with Suzy compared with their own experiences with follow-up care for SUD and shared recommendations for improvements to the chatbot. Concurrent think-aloud was used to understand what participants were thinking as they interacted with Suzy [46].

#### Measures

##### Background Characteristics

Health care professionals reported their gender and race/ethnicity, and patients reported their age, gender, and race/ethnicity. Patients also reported their technology savviness through a single question: “How tech savvy would you rate yourself, from 1 to 10?” [47].

##### Feasibility

The study team evaluated feasibility based on patients’ ability to access and use Suzy to perform and complete the predefined set of usability testing tasks.

##### Acceptability

The team recorded qualitative feedback during usability testing to evaluate acceptability. They also administered a survey that included a Net Promoter Score (NPS; [48]) question for users to rate the likelihood of recommending the chatbot to others. The NPS ranges from −100 to 100.

##### Usability

The survey also included 2 usability measures. The positive System Usability Scale (SUS; [49]) is a 10-item, 5-point Likert scale survey that indicates general usability, with scores ranging from 1 to 100. Participants also completed the Single Ease of Use Question (SEQ; [50]) for each usability task to indicate “how difficult or easy was the task to complete,” with possible scores ranging from 1=very difficult to 7=very easy.

#### Data Analysis

Descriptive statistics summarized participant sociodemographic characteristics, background information, and study measures, including means and SDs for continuous variables and Likert scales, and proportions and sample sizes for categorical measures.

### Phase 3: LLM-Based Chatbot Development

#### Rationale for LLM Development

LLM technology advances that occurred as the study team was completing usability testing with Suzy (ie, the public launch of ChatGPT; OpenAI) prompted them to incorporate LLMs into Suzy’s design. The LLM-based chatbot was built using an open-source platform developed by Dimagi Inc called Open Chat Studio (OCS), which interfaces with the LLMs used for this project and Dimagi Inc’s commercially available HIPAA-compliant and System and Organization Controls 2 (SOC 2)–certified platforms [51]. Using a combination of evidence-based source information and custom-engineered “prompts,” the LLM redesign enabled Suzy to engage users in dynamic, nonscripted conversations to address their substance use recovery journey in a more user-centered, contextualized way. LLMs also expanded the ways in which users could engage with Suzy and allowed Suzy to adapt to changing user information and needs (eg, dynamic appointment reminders versus static appointment reminders bound by fixed scripts and manual triggers). While results from phase 2 suggested that navigation and use of the rule-based chatbot were acceptable, respondents also noted potential limitations of a menu-driven tool, particularly for individuals who may be struggling to clearly identify their needs in the moment.

#### Co-Design

Following human-centered design principles, the study team co-designed the adapted LLM-based chatbot with 5 PRCs working in SUD treatment clinics who were recruited through professional networks from a major tertiary care hospital in Boston, Massachusetts, from July to September 2024. Four sequential co-design sessions were conducted with PRCs to address the following design objectives: (1) articulate current PRC workflows and patient interactions and discuss potential activities that could be supported by the LLM-based chatbot; (2) engage co-designers in a guided walkthrough of the prototype and collect impressions; (3) assess content usefulness, comprehensiveness, and related attributes; and (4) assess usability and user experience. All PRCs were given access to an LLM-based chatbot prototype for testing during the co-design period. Sessions were led by user experience–trained co-authors (YXH and DML), who also led qualitative interviews in phases 1 and 2. Co-designers provided continuous feedback across the 4 sessions on the LLM adaptation of proposed features informed by phase 2 feedback (eg, goal setting, appointment reminders, check-ins). They also provided feedback on new LLM-specific design aspects, such as Suzy’s persona (eg, language understandability and accessibility, tone), performance (eg, length of text responses, response time), and trusted content and local resources to be incorporated into retrieval-augmented generation. Content included local support groups and recovery programs recommended by PRCs that may not otherwise be up-to-date or accessible to the LLMs.

#### Expert Validation

Next, 2 coauthors (YXH and DML) solicited expert usability review and safety validation of Suzy’s responses. They convened a multidisciplinary expert panel of 7 study team members, including coauthors with expertise in clinical care, SUD, and behavioral intervention research (WSC, DS, JMS, and LG). Panelists tested, reviewed, and evaluated Suzy’s responses in 2 rounds of testing to inform modifications to Suzy’s prompt engineering between November 2024 and January 2025.

In the first round, panelists were provided access to Suzy and independently tested its performance across 8 predefined test scenarios representing typical use cases. The test scenarios and their respective criteria are described in Multimedia Appendix 2. Panelists were instructed to test Suzy as a patient receiving SUD treatment and to provide qualitative feedback on Suzy’s performance and the content of its responses based on predefined criteria. Panelists submitted their perceptions of the experience and recommendations, including issues related to Suzy’s technical performance (eg, system nonresponsiveness, operating system–related requirements to enable message exchanges, nonfunctional hyperlinks to community resources, message length) and the content of conversations between panelists and Suzy. Content evaluation encompassed Suzy’s adherence to the scope and purpose of addressing substance use, as well as responses to other topics such as sex-related content, how to obtain concert tickets, and appropriate handling of risky user messages. Responses were reviewed and prioritized by Suzy’s developers (including co-author DW), with technical performance–limiting issues assigned the highest priority, followed by content-related issues. Comments requiring further clarification or expert input were discussed in follow-up meetings. For example, panelists noted Suzy’s tendency to prematurely assume user intent. Discussion led to consensus that this was acceptable for some queries, such as referring users to preexposure prophylaxis services when a user asks Suzy to “recommend community resources if I want to take a pill to prevent HIV.” Otherwise, there was agreement that Suzy should provide follow-up responses to gather more information about the user’s situation and deliver a more relevant response.

The goal of the second round of testing was to inform refinements to Suzy’s routing architecture and safety handling based on expert feedback following initial validation, with particular attention to safety and accuracy. During the first round of testing, the study team identified limitations in the performance of a single, general-purpose routing node responsible for directing user messages to downstream chatbot functions, including safety-related pathways. Despite iterative adjustments to routing instructions and experimentation with pretrained (ie, non–fine-tuned) base LLMs, alignment between router outputs and expert panel judgments regarding safety routing remained suboptimal. Accordingly, the second round of testing was intentionally structured to generate labeled examples of expert safety determinations, which were then used to fine-tune a dedicated triage model. This fine-tuned model was subsequently deployed as an initial routing node to classify incoming messages and direct them either to a safety pathway, a clarifying (dig deeper) pathway, or to the existing general router for task-specific handling. As part of this revised architecture, the study team devised multiple strategies to mitigate chatbot risks for the target population, including a dedicated chatbot safety node with a “safety disclaimer” that includes scripted information on how to reach human support if and when needed.

Expert opinion combined with curated scenarios provided an evaluation approach to assess nuanced safety concerns that would have been difficult to capture using automated evaluation approaches [52]. Here, 4 expert panelists (coauthors WSC, DS, JMS, and LG) each reviewed a different set of 50 LLM-generated chatbot messages created from real-world examples of queries with varying levels of risk from patients struggling with SUD, including potential suicidal ideation. Multimedia Appendix 3 provides examples of test-set messages. Expert panelists responded *yes*, *no*, or *maybe* to the question, “Should Suzy respond with a Safety Disclaimer?” Qualitative feedback was encouraged when experts felt a response warranted further explanation. This feedback was reviewed and used by the chatbot developers to refine prompts and reduce risk. All expert feedback was collected independently and then shared nonanonymously on a design tracker to foster open discussion and discourse on items with notable disagreement. Expert responses and qualitative comments were reviewed by the chatbot developers to improve the chatbot’s ability to distinguish between risk levels and enhance responsive messaging.

#### LLM Platform

**Figure 2:**
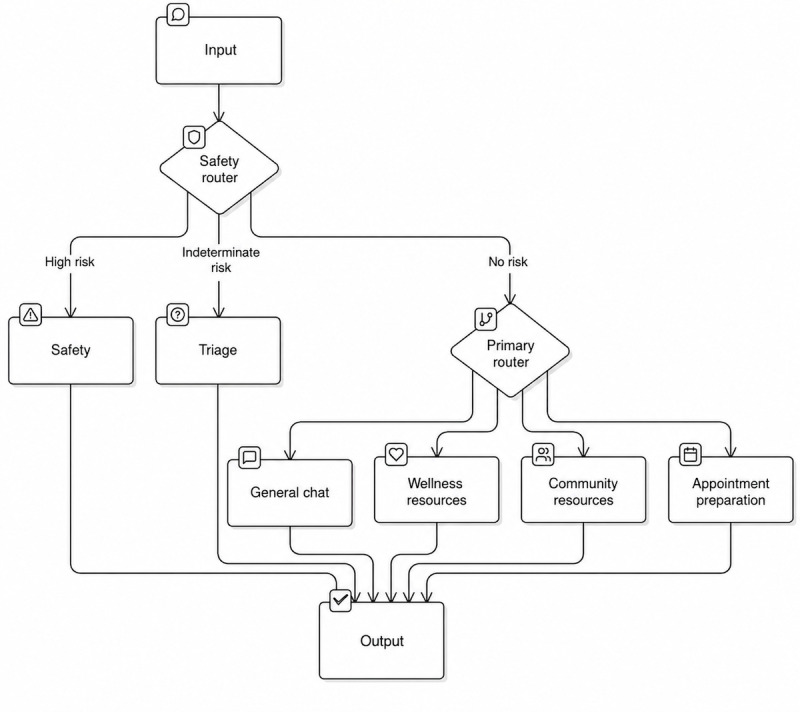
Chatbot pipeline architecture after large language model integration. When a user submits a message, the chatbot first performs a safety check. Messages that signal potential urgent risk are routed to a dedicated safety pathway, while ambiguous cases undergo triage review. Messages deemed safe are forwarded to the main system, which directs them to appropriate support modules, such as general conversation, wellness resources, community resources, or appointment preparation assistance. This layered architecture enables flexible dialogue while maintaining robust safety safeguards.

Compared with interacting with publicly available LLMs, OCS allowed us to implement a multiprompt architecture and incorporate additional safety layers for this target population using application programming interface integration with the LLMs determined to perform optimally for this chatbot build (OpenAI’s GPT-4o, GPT-4.1, and a custom fine-tuned GPT-4o safety model) [51]. OCS is a platform in development that has been used to support LLM-based chatbots for multiple use cases, from providing researchers with a way to query large data repositories to supporting social and behavioral change interventions. While limited in features during this project period to support what might be needed to make Suzy more robust and fully implementable, such as auto-graded evaluations and full end-to-end encryption, OCS was selected to build and test this chatbot prototype because it is easily configurable with Dimagi Inc’s case management and messaging platforms used to support other components of this system. Further, Dimagi Inc has a zero data retention agreement with the LLM provider (OpenAI), supported by a high-level privacy policy ensuring that user data are not stored, recorded, or logged on the provider’s end after a task is completed. OCS uses a configurable pipeline in which messages flow through specialized nodes (eg, LLM nodes and router/decision nodes). As reflected in Figure 2, incoming text is first evaluated by a safety router; high-risk content is directed to a dedicated safety pathway, indeterminate-risk content is directed to a triage node, while low-risk content proceeds to a primary router that delegates to task-specific nodes such as general chat, wellness resources, community resources, or appointment preparation. This node-based orchestration introduces a small increase in end-to-end latency relative to a single-prompt approach, but it substantially improves safety triage, consistency in instruction execution, and overall reliability.

Critically, with the LLM-based approach, we incorporated additional features to address accuracy and safety in anticipation of “hallucinations” and potential safety concerns in the absence of real-time human review. We designed the chatbot to prioritize safety and accuracy by including elements such as disclaimer language reminding users that Suzy is a chatbot and may make errors, and a “safety router” (as shown in Figure 2) to assist with triaging user inputs. This enables Suzy to respond immediately with safety messaging, including emergency contact information, if the input is determined to be risky (eg, suggests self-harm or harm to others), or to follow-up with clarifying questions to determine the level of risk. The chatbot’s sensitivity to risky inputs was informed by feedback from the content expert panel review of test user inputs, as described in the “Methods” section.

## Results

### Participant Background Characteristics

One-third of the 12 health care professionals who participated in phase 1 formative work identified as male (n=4), nearly two-thirds identified as female (n=7), and 1 health care professional identified as nonbinary. One-third of the health care professionals also reported Hispanic/Latino ethnicity (n=4). Among those who reported non-Hispanic ethnicity, race was reported as African American or Black (n=2, 17%), Asian (n=3, 25%), White (n=1, 8%), multiracial (n=1, 8%), or not reported (n=1, 8%); 5 (42%) health care professionals reported more than 7 years of experience working with patients with SUD.

Of the 14 patients who participated in phase 1 formative work or phase 2 usability testing, most identified as male (n=12, 86%), 1 (7%) identified as female, and 1 (7%) identified as nonbinary. More than half of patients identified as Hispanic/Latino (n=8, 57%), with the remaining non-Hispanic patients reporting their race as Black/African American (n=4, 29%), White (n=1, 7%), or multiracial (n=1, 7%). As many as 9 (64%) patients were between 18 and 39 years of age, with the remaining 5 (36%) between 40 and 69 years of age. A total of 10 (71%) patients reported having attended college, including 2 patients who completed a bachelor’s degree and 2 patients who completed a doctorate. One participant reported attending high school without receiving a diploma, 2 participants graduated from high school, and 1 participant received trade or vocational training. Most patients (n=13, 93%) reported use or misuse of 1 or more substances within 3 months before participation, and 4 (29%) reported use or misuse of 5 or more substances within this time frame. The most common substances, reported by more than half of patients, were tobacco, alcohol, cannabis, and methamphetamine; 2 (14%) reported experience with prescription opioids. The 8 patients who participated in usability testing reported an average perceived tech savviness score of 7 (SD 2.6) on a custom scale of 1 to 10, from low to high self-perceived tech savviness.

### Phase 1: Formative Work

**Figure 3:**
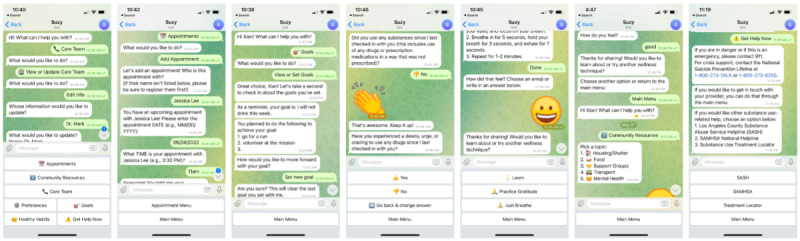
Sample screenshots of human dialogue with Suzy illustrating its core patient-support features (left to right): (1) manage and contact care team members; (2) create and receive appointment reminders; (3) set and review Specific, Achievable, Relevant, Time-Bound (SMART) goals; (4) check in about recent substance use and cravings; (5) learn urge-surfing and other wellness techniques; (6) search for community resources (eg, support groups, housing); and (7) connect with emergency resources (eg, 24-hour helplines).

Participants revealed gaps in support between SUD treatment clinic appointments that they perceived a chatbot could help alleviate. They discussed how a chatbot could facilitate patient engagement and linkage to care, address potential triggers of substance use cravings, and provide timely recovery support, particularly when immediate human support may be limited or unavailable. As summarized in Table 1, participant interviews also guided the development of Suzy by identifying the most appropriate uses of a chatbot for primary care patients with moderate risk of SUD and by prioritizing 7 chatbot features to engage patients with SUD: (1) health care team contact management (ie, contact information and facilitation via smartphone and email hyperlinks); (2) appointment reminders (ie, creating and receiving self-reminders for clinic appointments related to SUD treatment); (3) goal setting (ie, setting and reviewing Specific, Measurable, Achievable, Relevant, and Time-Bound [SMART] health-related goals); (4) check-ins about recent substance use and cravings; (5) cravings management (ie, urge-surfing and wellness technique instructions); (6) community resource information (eg, support groups, housing); and (7) emergency resources (ie, 24-hour help hotlines). Figure 3 shows example smartphone screenshots of rule-based Suzy-human interactions that reflect these 7 features.

**Table 1:** Chatbot features informed by phase 1 qualitative interviews with health care providers and patients, along with illustrative quotations.

Features	Quotations
Manage contact information for the care team	“Because through the course of time, I lost the numbers that they had to contact. They actually sent me letters trying to-- yeah, we were trying to contact you and stuff like that. No, they did their part. That's why, like I said, I got a phone, and I called them back, and I wanted to [get] back on track. Oh no, they did everything they were supposed to do, I will say. They tried to stay up on me, but-- especially after I started getting high.” [Patient]
Appointment reminders	“I could see a chatbot being helpful for an appointment reminder. If they do get an appointment to do an intake or whatever to ensure that that participant-- like an automated beforehand being like, ‘Here’s your appointment. Do you need transportation? Here’s a referral for that.’ Those kinds of things that will eliminate the barrier. The first barrier is just calling, right?” [Health care provider]
SMART^a^ goals	“So we have a loose protocol for goal setting...using motivational interviewing to help patients identify their goals, keeping the goals simple.” [Health care provider]
Substance use check-ins	“Even if it’s just a text that says, ‘Hey, just checking in today. Time for your-- how are you feeling?’ Sometimes a lot of these patients do tell me that they feel like they're kind of unsupported and alone. And so even though this is not a real person, I think it’s just that the fact that something, someone is reaching out to them in some aspect is helpful.” [Health care provider]
Urge surfing and wellness	“...something that they would ask their therapist is, ‘How do I cope with this if my anxiety is so bad and I want to use right now?’...the chatbot could have some direction in that, but probably not as intense as the therapist would.” [Health care provider]
Community resources	“And then also maybe even referrals so if they say, ‘I need assistance with-- I need a referral,’ then the chatbot can ask what they need help with, and then maybe it can kind of direct them where they can go.” [Health care provider]
Emergency resources	“If you’re feeling particularly low, the chatbot could maybe help provide the number or make a direct call to [the therapist].” [Patient]

^a^SMART: Specific, Measurable, Achievable, Relevant, and Time-Bound.

### Phase 2: Feasibility, Acceptability, and Usability Testing

#### Feasibility

Findings from this small pilot usability sample are presented below. All but 1 patient who participated in usability testing successfully used Suzy to complete the 12 usability testing tasks and complete the study. One participant did not complete the final 2 tasks—a breathing exercise and a trivia question—due to personal time constraints that prevented them from remaining for the full duration of the usability session. While the time required to complete a given task varied, it was consistent with task complexity, with average task times ranging from 49 seconds to find a helpline to 3 minutes and 21 seconds to enter goals.

#### Acceptability and Usability

Quantitative results from this small pilot sample are summarized in Table 2 and indicate high levels of preliminary acceptability and usability. The average SUS and NPS scores of 93 and 63, respectively, were well above industry benchmarks of 68 [53] and 30 for digital health apps [54], respectively. Seven participants made at least one usability error, most commonly the initial selection of an incorrect menu button to complete a given task, and 3 participants misinterpreted at least one task by responding other than instructed. However, the SUS and NPS findings indicate that, overall, Suzy was acceptable, participants were satisfied with its use, and were likely to recommend it to others. The average SEQ rating of 6.5 is close to the maximum value of 7 and indicates that the 12 commonly performed usability tasks were, on average, perceived as easy to complete.

**Table 2:** System Usability Scale, Net Promoter Scorea, and Single Ease of Use Question descriptive statistics for the 8 patients who participated in usability testing sessions.

Measure	Score	Possible range	Industry benchmark
System Usability Scale, mean (SD); median	93 (11.7); 97.5	1 to 100	68
Net Promoter Score	63	–100 to 100	30
Single Ease of Use Question, mean (SD); median	6.5 (–0.5); 6.5	1 to 7	N/A

^a^Difference between the percentage of respondents who give a score of 9 or 10 (promoters) and the percentage who give a score of 6 or lower (detractors).

Qualitative findings summarized in Table 3 align with the favorable quantitative results for Suzy. Participants reported that Suzy’s tone and language were approachable and understandable, and found the menu-based navigation easy to use. They also noted that Suzy provided a nonjudgmental platform to share information they might otherwise hesitate to disclose in human-to-human interactions. Multiple participants reported that Suzy could help improve overall wellness goals. Features such as goal-setting exercises, quizzes, and automated periodic check-ins about recent substance use were seen as a more immediate outlet to express feelings or “vent.” Participants viewed Suzy as a helpful resource that could be used in conjunction with conversations with care team members, particularly after the first 30 days of recovery and into later stages of SUD recovery. Features such as a repository of local community resources and emergency helplines, urge-surfing and wellness techniques, and support for self-reflection and accountability were also considered beneficial (see Table 1). Participants highlighted that human support is not always available when needed, and Suzy was regarded as a tool that could help fill gaps in support. However, participants also emphasized that human support is irreplaceable and that they relied on multiple care team members, including counselors, case managers, social workers, and sponsors, to manage their substance use.

**Table 3:** Themes and illustrative quotations from qualitative patient feedback during phase 2 usability testing organized by positive and negative aspects of using the chatbot.

Themes			Quotations
**Positive themes**			
	Nonjudgmental tone and language	“I did initially feel like, ‘Oh, I let Suzy down’ because I didn’t keep up with what I said I would. But how she (Suzy) reacted, I guess, made me feel comfortable that I can...change my habits.”	
	Case management and goal setting	“This is a great resource because it helped me case manage myself. If there’s anything that I worked on with my case manager or my therapist...I could set it on here and it could remind me throughout the day for my goals.”	
	Check-ins	“The usual things that I would do normally [referring to check-ins and appointment scheduling, it would be cool to use the chatbot for.”	
	Accountability and self-reflection	“When it comes down to it, that beautiful thing about the bot is that it’s very self-reflective without you even knowing it...It’s self-reflective because it holds you accountable to yourself.”	
	Local resource referral	“And then also the whole location thing, like if I’m out of town and I decide to use Suzy, to be able to have a way to enter a zip code or a city that I’m staying in particularly during that time to help find local resources, not just on a home base from my address. Sometimes I’m not at home.”	
	Filling gaps in support during off hours	“Suzy is great because sometimes my case manager and therapist don’t respond at 10 o’clock at night, and that’s the worst part...sometimes these events or situations happen late.”	
**Negative themes**			
	Lack of lived experience	“I want to speak to somebody that has been in my shoes, who really, really understand why I feel the way I do, the reason where there’s tears falling from my eyes. I want to talk to somebody that knows that exact feeling and that desperation, and I don’t feel like you get that from a machine.”	
	Difficulty finding information	“I mean, because normally when you get an app, you click on something and you just kind of scroll through it, find what you need, rather than on this app, maybe if things were a little more straightforward with certain things...instead of having to click multiple things to get to what you’re looking for, maybe if there was a icon already for it.”	

There were some notable challenges reported by participants when using the rule-based chatbot. These included the need for more immediate and direct information or referrals to helplines, difficulty entering certain requested information (eg, care team member contact information), lack of direct appointment support and other similar tasks that would likely require another app, and difficulty with goal setting. Participants appreciated the menu-based response options of the rule-based chatbot, but 1 participant noted that “if it (Suzy) can come out more human-like, yeah, I would love to use it.” Another participant suggested that voice-enabled chat would also be helpful. LLM integration addressed several of the key challenges reported by participants. For example, it provided greater flexibility for participants to schedule appointments, set goals, and engage with the chatbot using more natural, text-based responses.

### Phase 3: LLM Development

Co-design work with PRCs in early phase 3 suggested that certain functionalities, such as SMART-based goal setting, may be less useful and potentially confusing for patients who follow different goal-setting paradigms. Care team information also appeared less useful and potentially redundant, as patients may already have contact information stored on their smartphones, and this information would need to be current and regularly updated to provide meaningful utility.

Experts reported varying levels of risk tolerance and strategies for triaging potential self-harm and suicidal ideation. However, a more conservative approach was adopted, based on expert feedback, to determine which of the following 3 nodes a user would be directed to: (1) provide an immediate safety response to a risky user message; (2) provide a response to a nonrisky user message; and (3) a “dig deeper” node to respond to messages with ambiguous intent.

Table 4 compares the key features of the rule-based chatbot deployed for usability testing with those of the LLM-based chatbot designed for future testing. In transitioning to the LLM-based chatbot, 1 major change was the replacement of button-based, short key-phrase interactions with dynamic, natural language support. Features such as check-ins on wellness and cravings, wellness and urge-surfing techniques, and connections to community resources were retained and adapted for LLMs. For example, co-design work and expert panel discussions indicated that individual goal-setting activities may vary considerably, and the ability to set goals beyond or instead of SMART goals may be preferred. Another new function in the LLM-based chatbot is appointment preparation, linked to appointment reminders, in which Suzy asks whether the patient would like support in preparing for an upcoming appointment by prioritizing discussion topics and role-playing conversations. Moving beyond scripted dialog to an LLM-based dialog enabled the addition of features that can be more tailored to users in the moment (eg, dynamic reminders, location-based resources) and the expansion of existing features (eg, more flexible, user-led goal setting not limited to SMART goals) to better meet participant needs. Instead of navigating menus to find helplines, participants can now type requests directly to Suzy to obtain the resources they need. Other new procedural functions (eg, chatbot and safety disclaimers) that are only implementable with the LLM-based chatbot are listed in Table 4.

**Table 4:** Comparison of features between rule-based and large language model–based chatbots.

Features	Rule based	Large language model
Select buttons containing text responses (eg, “yes” or “no”) to interact with the chatbot	Yes	No
Check-ins on wellness and cravings	Yes	Yes
Guidance on how to alleviate cravings (eg, urge surfing techniques)	Yes	Yes
Connect user with hotlines or helpful community/clinic resources	Yes	Yes
Practice wellness techniques	Yes	Yes
Use of HIPAA^a^-compliant, SOC-2^b^–certified messaging platform (SureAdhere vs previous Telegram platform)	No	Yes
Natural language support	No	Yes
Ability to support user-led goal setting related to substance use (eg, a goal to exercise more)	No	Yes
Dynamic appointment reminders based on user preferences	No	Yes
Completion of chatbot disclaimer required before chatbot use to clarify that the chatbot is not human and its limitations	No	Yes
Safety layers (eg, safety routers/nodes to assist with triaging of risky user inputs, directing the user to immediately seek human help if the chatbot detects user responses that indicate potential self-harm or harm to others)	No	Yes

^a^HIPAA: Health Insurance Portability and Accountability Act.

^b^SOC 2: System and Organization Controls 2.

## Discussion

### Principal Findings

In this study, we addressed the goal of developing a health coach aide chatbot, Suzy, to support patients in SUD recovery between treatment visits by (1) developing and validating a rule-based chatbot prototype for feasibility, acceptability, and usability testing; and (2) adapting the chatbot design using LLMs to enhance user experience and potential impact. Quantitative measures such as the SUS indicated favorable usability for the rule-based version of Suzy, consistent with qualitative findings. Positive patient feedback highlighted Suzy’s functional features, including goal setting, techniques to alleviate substance use urges, and service referrals, as well as its ability to create engaging user experiences (ie, hedonic qualities), such as helping patients express their feelings and engage in self-reflection. The feasibility, acceptability, and usability of Suzy as a health coaching tool for patients were consistent with prior studies that deployed chatbots for screening and intervention delivery [21,24,25].

Our study adds to prior AI research in addiction medicine by examining the role of a chatbot explicitly designed to complement PRC workflows and support patients between office-based SUD recovery visits. We describe a developmental process that can be used to design other patient-facing chatbots that play a critical role as part of the clinical care team to support and empower patients in long-term substance use recovery, while potentially reducing clinical work burden. The role of a chatbot embedded within a clinical care team aligns with a “more-than-human care” model that affirms the crucial role of humans in anchoring care, while recognizing the potential for chatbots to streamline and enhance service delivery beyond what humans alone can provide [55]. This perspective was echoed in participant feedback, which emphasized the importance of human case managers and therapists, while noting that they are not always reachable after work hours. Chatbots have the potential to help health care providers make their services more accessible to patients outside of clinic settings and office hours. Participant feedback was also consistent with the model of supportive accountability in digital health interventions [56]. Chatbot interactions can further support tailored intervention delivery. For example, clinicians could use AI to analyze patient transcripts—traditionally from conversations with human counselors, but also from chatbot interactions—and pair these data with patient outcomes to refine evidence-based practices [57].

Greater integration of Suzy with PRC services may have driven even higher levels of patient acceptability than were feasible or observed in this study. In a review of factors affecting the acceptability of chatbots in health care, patients’ acceptance of chatbots depended on the therapeutic relationship (working alliance) they had with their physicians in 27% of studies [58]. Despite the lack of chatbot integration into PRC workflows in this study, patients perceived chatbot coaching support as an extension of PRC support. Participants also reported being willing to share information with Suzy that they might be less likely to share with a human and perceived Suzy as “nonjudgmental,” a notion expressed in another study evaluating a chatbot embedded in an SUD treatment setting [21]. By contrast, a qualitative study of Australian adults receiving online alcohol and drug counseling services found that participants would share less information with a chatbot than with a human counselor [55]. It may have been more difficult for participants in that study to establish rapport and trust with the chatbot in an online environment. Further research into factors influencing acceptability is warranted, particularly in light of potential confounders across studies, such as increased societal familiarity with AI and chatbots between the earlier study of Australian adults and this study.

Cultural, social, and demographic factors shape SUD treatment access and engagement [59]. SUDs disproportionately affect individuals facing structural inequities, including housing instability, barriers to health care and digital access, and low technological literacy. Additionally, stigma related to SUD intersects with these factors in ways that influence patient trust in health care systems, including chatbot interventions and AI algorithmic risk across populations. These considerations underscore the importance of ongoing chatbot evaluation across diverse populations and the need to tailor chatbots using culturally responsive language and safety guardrails during development. Encouragingly, studies have shown that properly tailored chatbots can facilitate engagement, particularly among marginalized populations facing health care stigma and among individuals with low technological literacy [60].

To stay responsive to AI advances, our development process evolved iteratively. We initially focused on a rule-based chatbot to select the most robust option available at the time. This approach aligns with prior chatbot development in addiction medicine, where similar systems employed machine learning/AI algorithms to select responses from a closed library of predefined options [21]. Current discussions on chatbot innovation often center on LLMs, as reflected in the next phase of development in this study. Moving forward, we will continue to leverage LLM advances that balance Suzy’s ability to engage in humanlike, appropriate conversations, minimize errors (eg, ensuring accurate service referrals), and maintain patient privacy. To support this balance, an emerging body of research is exploring the combined use of rule-based and LLM-based chatbots to deliver different components of health care interventions, such as sexual health interventions [61]. This approach leverages the strengths of both systems—the reliability of rule-based chatbots and the flexibility and more natural conversational abilities of LLM-based chatbots when appropriate. Ultimately, decisions regarding which AI and technology innovations to implement will be driven by the need to develop robust, scalable chatbots capable of operating effectively within large-scale health care systems [23].

Our development process highlighted real-world strategies for mitigating clinical, ethical, and operational risks during chatbot implementation in addiction medicine settings. First, chatbots designed to support patients in SUD treatment need to operate within clearly defined escalation pathways. We trained Suzy so that high-risk or ambiguous interactions (eg, patient responses indicating possible suicidal ideation) prompt it to direct patients to seek crisis services. Second, patient onboarding procedures are essential to ensure that users understand chatbot capabilities, limitations, privacy protections, and appropriate use cases. Study onboarding instructions guided patients to seek medical advice from clinical providers rather than from Suzy. Third, health care providers need to monitor chatbots on an ongoing basis and establish governance structures to mitigate ethical and operational risks. For example, we monitored patient interactions with Suzy throughout the study period, mirroring ongoing monitoring practices that would occur in a clinical setting outside of a research context. Finally, operational integration into existing workflows is crucial; without it, there is a risk of diffusion of responsibility and overreliance on automated chatbot support. Guidance can be drawn from other emerging AI tools in health care settings, such as ambient AI scribes that integrate with electronic health records to reduce physicians’ administrative burden while still requiring oversight [62,63]. Our study also prompted discussions among clinicians, PRCs, and developers regarding future implementation planning and staff training to accompany chatbot deployment in addiction medicine settings.

This study was limited by its dual exploratory aim to design and evaluate a novel chatbot to support individuals in substance use recovery and to adapt it using evolving LLM technology while maintaining its specific scope and purpose. Key limitations include a small sample of 14 patients recruited to evaluate the early, rule-based prototype. This sample size limits generalizability across patient populations affected by SUD. Although patients were diverse in terms of race/ethnicity and age, they were not diverse by sex; all patients identified as male except 1 female and 1 nonbinary patient. Nonetheless, the sample size was sufficient to inform early-stage development and future research. Another limitation is that the chatbot was adapted using a single LLM (GPT-4o), which may limit generalizability to other models. Since the finalization of the adapted version, newer models (eg, GPT-5 series) have emerged, offering faster processing, improved reasoning capabilities, including for health-related responses, and reduced hallucinations. The rapid evolution of LLMs necessitates more efficient and robust evaluation methods, which were still emerging at the time of this study. We reiterate that this study evaluated the feasibility, acceptability, and usability of a rule-based chatbot with patients. The LLM-based version was assessed only through expert co-design and safety review and is currently being pilot-tested with patients in medication treatment for opioid use disorder. This study does not make claims regarding clinical efficacy or reduction in substance use. While the findings suggest potential utility of an LLM-based chatbot, further research is needed to understand its implications in controlled, real-world settings before it can be considered for broader deployment.

As a continuation of this pilot work, we will further refine Suzy’s functionality and performance to better meet the needs of patients receiving SUD treatment. We are currently conducting a National Institutes of Health–funded study to evaluate the preliminary efficacy of Suzy with LLM integration in reducing substance use among patients receiving SUD treatment, with a more demographically diverse sample than in this study.

### Conclusion

AI-based chatbots have the potential to enhance the role of PRCs and clinical care teams in supporting patients receiving SUD treatment. Chatbots can help patients manage appointments, provide service referrals, and guide patients through coaching activities after hours and between clinic visits and contacts with PRCs. Generative AI innovations enable the deployment of LLM-based chatbots that mimic human conversation and address more complex patient inquiries than was possible with nongenerative AI chatbots. However, much remains unknown about the implications of human-chatbot interactions, with conflicting reports on social effects such as the need for human-to-human contact [64,65]. Additional research is needed to determine the safest and most effective use of LLM-based chatbots in health care, designed to support not only patients but also PRCs directly and within human-in-the-loop interactions. Health care system policy changes to support AI integration and infrastructure improvements may further enhance the support that chatbots can provide to PRCs and their patients. However, such integration requires the highest data safety standards and sustained trust in the health care system. Given the limited sample size of this pilot study, the findings are not generalizable beyond the study scope. Nonetheless, they can inform the design of future iterations of this chatbot and other LLM-based systems, contributing to a broader understanding of how such technologies can be safely leveraged to support patients in SUD recovery.

## Appendix

Multimedia Appendix 1 TRIPOD-LLM (Transparent Reporting of a multivariable prediction model for Individual Prognosis Or Diagnosis – Large Language Models) checklist.

Multimedia Appendix 2 Eight test scenarios and evaluation criteria used by the study team's expert panel to assess Suzy after large language model integration during the first round of testing. Experts were instructed to assume the role of a patient receiving substance use disorder treatment and to evaluate Suzy’s responses according to predefined acceptability criteria.

Multimedia Appendix 3 Eleven test-set message examples reviewed by the study team’s expert panel during the second round of testing to evaluate Suzy after large language model integration. Experts were asked to determine whether Suzy should include a safety disclaimer in response to each message by selecting “Yes,” “No,” or “Maybe.” Additional comments were encouraged to justify their selections.

## Abbreviations

HIPAA: Health Insurance Portability and Accountability Act
IRB: institutional review board
LLM: large language model
NPS: Net Promoter Score
OCS: Open Chat Studio
PRC: peer recovery coach
SEQ: Single Ease of Use Question
SMART: Specific, Measurable, Achievable, Relevant, and Time-Bound
SOC 2: System and Organization Controls 2
SUD: substance use disorder
SUS: System Usability Scale
TRIPOD: Transparent Reporting of a multivariable prediction model for Individual Prognosis Or Diagnosis

## Data Availability

Study data consist of formative interview transcripts and transcripts of developer test conversations and prompt iterations. Requests for data access should be directed to the corresponding author (WSC).
