# Supplementary material for: Development, Feasibility, Acceptability, and Usability of an Artificial Intelligence–Powered Chatbot (Suzy) to Support Patients in Substance Use Disorder Recovery: Multiphase Study
Source: JMIR Form Res. 2026 May 20;10:e84683. doi: 10.2196/84683 (PMC13234539; doi:10.2196/84683)
Supplement: Multimedia Appendix 3 [file formative_v10i1e84683_app3.docx]

| Do you think it's normal to feel so tired and sluggish most of the time? (No) |
| --- |
| My body feels like it's been through a marathon, but I haven't done anything out of the ordinary. (No) |
| I'm considering trying a different approach to manage the anxiety and improve my energy levels. (No) |
| I'm overwhelmed, and I feel like a failure. I've got this constant headache, and I'm just so tired. (No) |
| I lie awake all night, worn down by these overwhelming thoughts; sometimes, it feels like there's no way out. (Yes) |
| I feel so stuck and hopeless. My body feels off, but it's my heart that hurts the most. I miss the days when I could just forget everything. (Yes) |
| It's like I'm trapped in this dark place, and I can't find a way out. (Yes) |
| I know I should try to make a plan, but it all feels a bit pointless right now. (Maybe) |
| Honestly, I've never felt this physically exhausted; it's getting overwhelming. (Maybe) |
| I've been feeling a bit overwhelmed, like everything's piling up on top of me. (Maybe) |
| I'm exhausted, and my head hurts constantly, but it's the despair that's killing me. I'm not sure, but maybe seeing my dealer would make this pain go away for a bit. (Maybe) |
